# Improving Diagnostic Performance for Head and Neck Tumors with Simple Diffusion Kurtosis Imaging and Machine Learning Bi-Parameter Analysis

**DOI:** 10.3390/diagnostics15060790

**Published:** 2025-03-20

**Authors:** Suzuka Yoshida, Masahiro Kuroda, Yoshihide Nakamura, Yuka Fukumura, Yuki Nakamitsu, Wlla E. Al-Hammad, Kazuhiro Kuroda, Yudai Shimizu, Yoshinori Tanabe, Masataka Oita, Irfan Sugianto, Majd Barham, Nouha Tekiki, Nurul N. Kamaruddin, Miki Hisatomi, Yoshinobu Yanagi, Junichi Asaumi

**Affiliations:** 1 Department of Oral and Maxillofacial Radiology, Graduate School of Medicine, Dentistry and Pharmaceutical Sciences, Okayama University, Okayama 700-8558, Japan; pokk3oeo@s.okayama-u.ac.jp (S.Y.); ya7@okayama-u.ac.jp (Y.Y.);; 2 Radiological Technology, Graduate School of Health Sciences, Okayama University, Okayama 700-8558, Japan; 3 Department of Oral Medicine and Oral Surgery, Faculty of Dentistry, Jordan University of Science and Technology, Irbid 22110, Jordan; 4 Department of Health and Welfare Science, Graduate School of Health and Welfare Science, Okayama Prefectural University, Okayama 719-1197, Japan; 5 Graduate School of Interdisciplinary Sciences and Engineering in Health Systems, Okayama University, Okayama 770-8558, Japan; 6 Department of Oral Radiology, Faculty of Dentistry, Hasanuddin University, Makassar 90245, Indonesia; 7 Department of Dentistry and Dental Surgery, College of Medicine and Health Sciences, An-Najah National University, Nablus 44839, Palestine; 8 Department of Oral Rehabilitation and Regenerative Medicine, Graduate School of Medicine, Dentistry and Pharmaceutical Sciences, Okayama University, Okayama 700-8558, Japan; 9 Department of Dental Materials, Faculty of Dentistry, Hasanuddin University, Makassar 90245, Indonesia

**Keywords:** head and neck tumors, mean kurtosis, simple diffusion kurtosis imaging, magnetic resonance imaging, apparent diffusion coefficient value, diffusion kurtosis imaging, machine learning, bi-parameter analysis, gradient boosting, differential diagnosis of benign and malignant

## Abstract

**Background/Objectives:** Mean kurtosis (MK) values in simple diffusion kurtosis imaging (SDI)—a type of diffusion kurtosis imaging (DKI)—have been reported to be useful in the diagnosis of head and neck malignancies, for which pre-processing with smoothing filters has been reported to improve the diagnostic accuracy. Multi-parameter analysis using DKI in combination with other image types has recently been reported to improve the diagnostic performance. The purpose of this study was to evaluate the usefulness of machine learning (ML)-based multi-parameter analysis using the MK and apparent diffusion coefficient (ADC) values—which can be acquired simultaneously through SDI—for the differential diagnosis of benign and malignant head and neck tumors, which is important for determining the treatment strategy, as well as examining the usefulness of filter pre-processing. **Methods:** A total of 32 pathologically diagnosed head and neck tumors were included in the study, and a Gaussian filter was used for image pre-processing. MK and ADC values were extracted from pixels within the tumor area and used as explanatory variables. Five ML algorithms were used to create models for the prediction of tumor status (benign or malignant), which were evaluated through ROC analysis. **Results:** Bi-parameter analysis with gradient boosting achieved the best diagnostic performance, with an AUC of 0.81. **Conclusions:** The usefulness of bi-parameter analysis with ML methods for the differential diagnosis of benign and malignant head and neck tumors using SDI data were demonstrated.

## 1. Introduction

Head and neck malignancies account for only about 2.3% of all malignancies in Japan [1], and odontogenic tumors are even rarer among oral and maxillofacial lesions, accounting for about 1.8% [2]. The pre-treatment diagnosis of benign or malignant head and neck tumors through imaging is important in determining the subsequent course of treatment [3,4]. Diffusion-weighted imaging (DWI) of MRI is considered useful for the diagnosis of head and neck tumors in routine clinical practice [5,6]. In particular, apparent diffusion coefficient (ADC) maps, which represent the ADC values calculated through DWI, are commonly used in daily clinical practice. Furthermore, diffusion kurtosis images (DKIs), which visualize the mean diffusion kurtosis (MK) values calculated through DWI, can capture the tissue microstructure in detail [7,8,9,10]; however, they are still not widely used in routine clinical practice, due to the long imaging time and the fact that dedicated imaging software is not standard in MR systems.

We have previously developed a simple diffusion kurtosis imaging (SDI) method using short-time imaging and general-purpose image processing software [11], and showed that it is useful for the diagnosis of head and neck tumors [12] and head and neck cyst diseases [13]. SDI allows DKI and ADC maps to be acquired simultaneously in a short time using triaxial imaging and three b-values, which are the imaging conditions for ADC maps in routine clinical practice. In SDI, the inhomogeneity of MK values due to the small amount of collected information poses a challenge, where pre-processing with a smoothing filter in DWI has been shown to improve the inhomogeneity of MK values as well as the tumor diagnostic ability [12,13,14].

It has recently been shown that the diagnostic performance of DKI can be improved through multi-parameter analysis in combination with another modality, such as DWI, intravoxel incoherent motion, or positron emission tomography, rather than DKI alone [15,16,17]. A key advantage of SDI is that ADC and MK values can be acquired simultaneously at the pixel level within a short time. The first objective of this study was to investigate, using machine learning (ML), whether bi-parameter analysis using both ADC and MK values obtained via SDI improves the diagnostic performance of benign or malignant head and neck tumor imaging, compared to that using single images. The second objective was to determine whether pre-processing with a smoothing filter would further improve the diagnostic performance.

There have been no previous reports of bi-parameter analysis of SDI using ML, and this is the first report showing that SDI can be used for ML-based bi-parameter analysis to aid in the differential diagnosis of benign and malignant head and neck tumors.

## 2. Materials and Methods

### 2.1. Patients

The study included 32 patients who underwent head and neck MRI as part of routine medical care for suspected head and neck mass lesions between 25 March 2020 and 30 May 2023, and who were pathologically diagnosed with neoplastic lesions. Exclusion criteria included cases of metastatic cancer (2 patients), tumors with a small diameter of less than 10 mm (38 patients), and cases with artifacts on lesion images (8 patients). All patients gave written informed consent for MR imaging. The study was conducted in accordance with the guidelines of the Declaration of Helsinki and was approved by the Ethics Committee of the Okayama University Graduate School of Medicine, Dentistry and Pharmaceutical Sciences and the Okayama University Hospital, 2209-014.

### 2.2. MRI System and DWI Sequence

The MRI systems used were a 3T MAGNETOM Prisma, 3T MAGNETOM Verio, 3T MAGNETOM Skyra, and 1.5T MAGNETOM Aera (Siemens Healthcare, Erlangen, Germany) with head and neck coils. The number of patients picked up by each device is as follows: Prisma 9; Verio 5; Skyra 11; Aera 7. The imaging sequence used to obtain ADC maps in routine clinical practice was used to obtain DWI with three axes and three b-values. Typical imaging parameters were used, as follows: b-values of 0, 400, and 800 s/mm^2^; slice thickness = 3 mm; repetition time (TR)/time to echo (TE) = 6990 to 12,300/55 to 84 ms; field of view (FOV) = 200 mm × 200 mm; gap = 4 mm; matrix = 140 × 140, 128 × 128, and 126 × 126; and bandwidth = 990 Hz/pixel. The average acquisition time for DW images was 230 s. In addition to DWI, contrast-enhanced T1-weighted, T1-weighted, T2-weighted, and T2 STIR-weighted images were obtained as part of routine clinical practice.

### 2.3. Pre-Processing with Smoothing Filter for DWI

Since smoothing by filter pre-processing of DWI has been reported to improve diagnostic accuracy by homogenizing MK values [14], filter pre-processing was performed as follows. The Gaussian filter process was used as the pre-processing filter (with σ = 0.5) using the ImageJ 1.51h (U.S. National Institutes of Health, Bethesda, MD, USA) image analysis software [14].

### 2.4. Creation of DKI and ADC Maps with SDI

To generate the DKI and ADC maps, we used the previously reported SDI [11,12,18]. Specifically, the DKI and ADC maps were created simultaneously by calculating the MK and ADC values for each pixel using the SDI software (v1.0) [11,12,18], utilizing the three b-value DWIs used to create ADC maps in routine clinical practice. This software uses macro programs in ImageJ and Microsoft Excel 2019 (Microsoft, Redmond, WA, USA).

**Figure 1:**
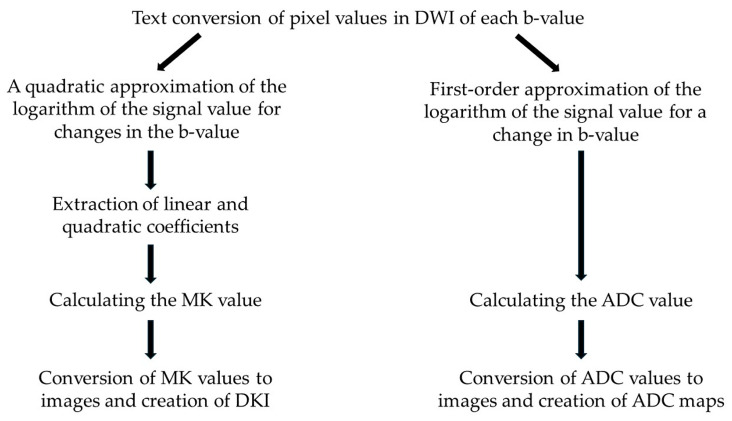
Overview of the DKI and ADC maps creation process. DWI, diffusion-weighted imaging; MK, mean kurtosis; DKI, diffusion kurtosis images; ADC, apparent diffusion coefficient.

Figure 1 shows the process of creating DKI and ADC maps.

To create the DKI, in each pixel of the three DWIs with b-values of 0, 400, and 800 s/mm^2^, the logarithm of each signal value was plotted on the vertical axis and the b-value on the horizontal axis, approximated by the quadratic function y = Ax^2^ + Bx + C, in order to obtain the quadratic coefficient A and the linear coefficient B. The MK value for each pixel was calculated using Equation (1). MK values were converted to images using ImageJ to generate the DKI [11,18].

MK = 6A/(−B)^2^. (1)

To create the ADC map, at each pixel of the three DWIs with b-values of 0, 400, and 800 s/mm^2^, the logarithm of each signal value was plotted on the vertical axis and the b-value was plotted on the horizontal axis, approximated as a linear function with y = Ax + B and the ADC values were calculated as −A. ImageJ was used to convert the ADC values into images, and the ADC map was created.

### 2.5. Region of Interest (ROI) Setting and Pixel Count Evaluation

ROIs were defined by consensus among five radiologists (M.K., J.A., Y.S., Y.F., and S.Y.). The tumor ROI was defined on DWI with a b-value of 0 s/mm^2^ in the slice with the largest tumor area. T2-weighted STIR MRI was consulted as needed, in order to correct the position and shape of the ROI.

A permutation test was performed using R (v4.2.2), in order to compare the number of pixels contained within the ROI for each tumor by histological type (i.e., benign or malignant), with *p* < 0.05 considered statistically significant.

### 2.6. Evaluation of MK and ADC Values by Tumor Status Histology

MK and ADC values were extracted from all pixels within each ROI, and pixel data were merged and analyzed according to tumor status (i.e., benign or malignant). For each of the MK and ADC values, Mann–Whitney U-tests were performed using EZR (v1.61) to compare significant differences by benign versus malignant or by presence versus absence of filter pre-processing. The Fligner–Killeen test for homogeneity of variance was conducted using R in order to determine the significance of variance for MK and ADC values. *p* < 0.05 was considered statistically significant.

### 2.7. Obtaining AUC Values Using Conventional ROC Analysis for Diagnosis of Tumor Status

The MK and ADC values of all pixels within the defined ROI were extracted in each case, and the pixel data were integrated and analyzed separately for benign and malignant tumors based on the pathological diagnosis of each case.

To evaluate the diagnostic ability of MK and ADC values alone for benign and malignant tumors, receiver operating characteristic (ROC) curve analysis was used to calculate the area under the ROC curve (AUC). R and EZR were used for the statistical analyses.

Multivariate ROC analysis was performed using EZR to evaluate the diagnostic ability of bi-parameter analysis with MK and ADC values to differentiate between benign and malignant tumors.

The diagnostic ability of benign and malignant tumor status was judged as “very excellent”, “excellent”, “good”, “satisfactory”, or “unsatisfactory” with AUC of 1.0–0.9, 0.9–0.8, 0.8–0.7, 0.7–0.6, and 0.6–0.5, respectively.

### 2.8. Obtaining AUC Values Using ML ROC Analysis for Diagnosis of Tumor Status

#### 2.8.1. Software and ML Algorithms Used

Anaconda Python version 3.11.9 and the Python library (Python Software Foundation, Wilmington, DE, USA) were used for the study. Five supervised ML algorithms were used: Gradient boosting (GB), deep neural network (DNN), random forest (RF), support vector machine (SVM), and decision tree (DT).

#### 2.8.2. Data Set (Tables S1 and S2)

MK and ADC values for all pixels within the set ROI in each case were extracted, and pixel data were integrated by benign and malignant tumors, based on the pathological diagnosis of each case, and used as explanatory variables. The distinction between benign and malignant tumors based on pathological diagnosis was considered as the objective variable. The number of pixels used as data in this study was unbalanced: 5636 for benign tumors and 3910 for malignant tumors without smoothing filter pre-processing, and 5645 for benign tumors and 3913 for malignant tumors with smoothing filter pre-processing.

#### 2.8.3. Best Modeling and Validation Practices

**Figure 2:**
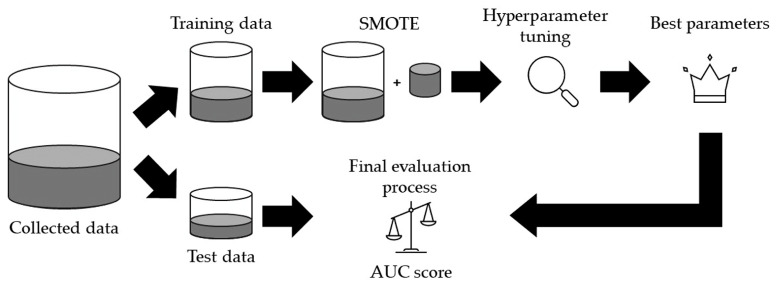
Overview of model building and validation process. SMOTE, synthetic minority oversampling technique.

Figure 2 shows an overview of the model building and validation process.

The data set was split using the train_test_split function in Scikit-learn version 1.4.2, with 80% as the training set and 20% as the test set. This split used stratified sampling to reflect the imbalance in the data. The synthetic minority over-sampling technique (SMOTE) was applied to correct the imbalance between the benign and malignant pixels in the training set. SMOTE is a technique that improves model training performance through synthesizing small-class samples to improve class imbalance and produce a balanced data set. For the DNN model, the data imbalance was addressed by adjusting the class weights.

To optimize the performance of the algorithm, hyperparameter tuning was performed using GridSearchCV. Optimization was performed using 5-fold cross-validation for DNN and RepeatedStratifiedKFold with 5 iterations of 5-fold cross-validation for GB, RF, SVM, and DT. The ROC-AUC score was used as the evaluation index. In the best model with the optimal hyperparameters, the final validation of the predictions against the test data was performed. In addition to AUC values, the accuracy, precision, recall, F1 score, specificity, Cohen’s kappa, and Matthews correlation coefficient (MCC) were calculated. The best GB models with and without filter pre-processing, as well as the GB’s best hyperparameters with the final validation results with and without filter pre-processing are shown in Codes S1, S2, and Hyperparameters S1, S2, respectively.

In the DNNs, we also introduced an early stopping method to suppress overlearning, and we applied batch normalization and probabilistic neuron deletion. In addition, dynamic learning rate adjustment was applied to stabilize model convergence.

### 2.9. Comparison of AUC Values for Diagnosis of Tumor Status

Significant differences between multiple AUC values obtained using conventional and ML methods were determined using the Delong test. R was used to determine the differences, and a *p*-value < 0.05 was considered statistically significant.

## 3. Results

### 3.1. Clinical Case Information

According to the eligibility criteria, 17 malignant tumors and 15 benign tumors were included in this study. Case information is listed in Table 1.

### 3.2. Comparison of ADC and MK Values in Benign and Malignant Histologic Types

The permutation test showed no significant difference in the number of pixels between benign and malignant histologic types. Therefore, in subsequent studies, the ADC and MK values of each pixel in the ROI of each tissue included in benign and malignant were analyzed together by benign or malignant status—the so-called pixel analysis [12].

**Figure 3:**
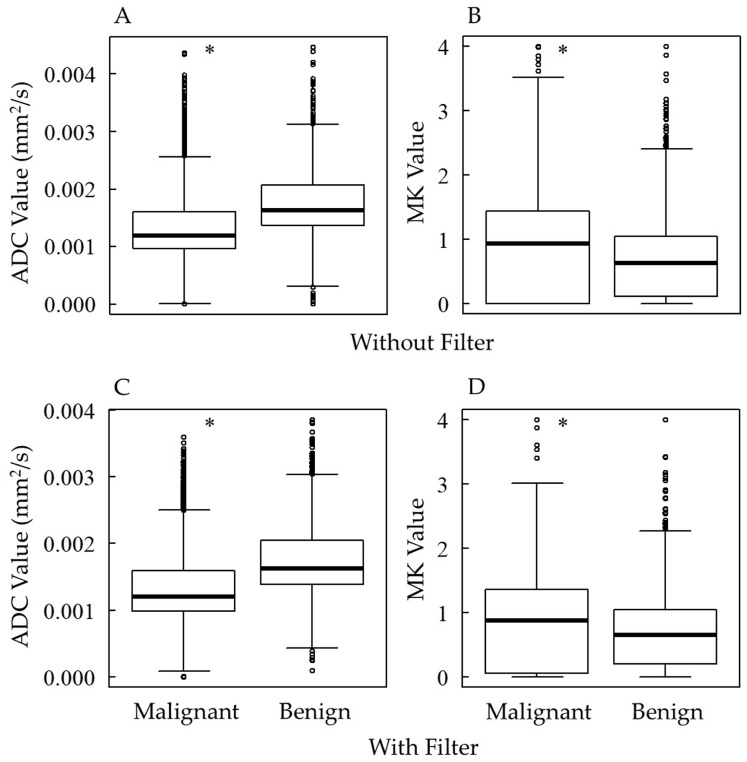
ADC and MK values in each histological type of tumors (i.e., benign and malignant): (**A**) ADC value without filter pre-processing; (**B**) MK value without filter pre-processing; (**C**) ADC value with filter pre-processing; and (**D**) MK value with filter pre-processing. ADC, apparent diffusion coefficient; MK, mean kurtosis. * *p* < 0.05 vs. benign. The dots in the figures indicate outliers.

Figure 3 shows the ADC and MK values for each benign and malignant tissue type. With and without filter pre-processing, malignant tissues had significantly lower ADC values and higher MK values than benign tissues. Table 2 shows the median ADC and MK values (Q1, Q3) with and without filter pre-processing. Filter pre-processing showed no significant differences in median ADC and MK values for both malignant and benign cases, while the variance of ADC and MK values for malignant and benign cases was significantly reduced, except for benign MK values.

### 3.3. Comparison of AUC Values Between ML and Conventional Methods

Table 3 summarizes the significant differences between the AUC values obtained when using ML and conventional methods. In the bi-parameter analysis using both ADC and MK values without filter pre-processing, the highest AUC value of 0.81 was obtained when GB was used as the algorithm, and the diagnostic performance was classified as “excellent”.

In the comparison between ML and conventional methods, the median AUC values of the five algorithms were all higher than those of the conventional method. In the comparison between each algorithm and the conventional method, three algorithms—namely, GB, DNN, and RF—had significantly higher AUC values than the conventional method and the other ML algorithms. There were no significant differences between these three algorithms.

### 3.4. Comparison of AUC Values Between Bi- and Single-Parameter Analyses

In the comparison between the bi- and single-parameter analyses of ADC and/or MK values, the median AUC values of the five algorithms were significantly higher in the bi-parameter analyses than in the single-parameter analysis, regardless of whether the algorithms were filter pre-processed or not.

Table 4 summarizes the significant differences in AUC values between bi- and single-parameter analyses for each of the three algorithms—GB, DNN, and RF—which showed significantly higher AUC values in the bi-parameter analysis than in the conventional method. For each of these three algorithms, the AUC values for the bi-parameter analysis were significantly higher than those in both of the single-parameter analyses, regardless of whether or not there was filter pre-processing.

**Figure 4:**
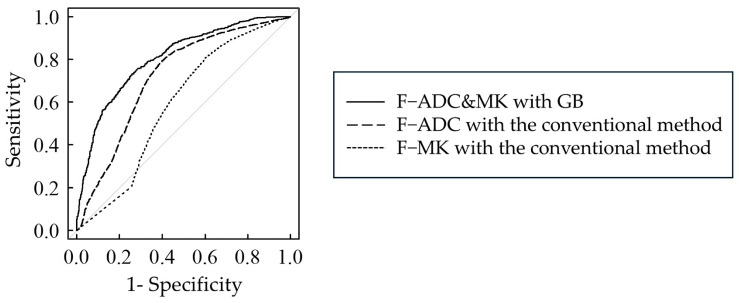
ROC curve based on ML and conventional methods. F−, without filter pre-processing; ADC&MK, bi-parameter analysis using ADC and MK; ADC, apparent diffusion coefficient; MK, mean kurtosis; GB, gradient boosting. Gray line indicates an AUC value of 0.50.

Figure 4 shows the ROC curves indicating the AUC values for each of the ADC and MK values using the conventional method, as well as the GB, which obtained the largest AUC value in the ML bi-parameter analysis. The bi-parameter analysis with GB (AUC 0.81) significantly improved the diagnostic performance when compared to the ROC curves for ADC alone (AUC 0.72) and MK alone (AUC 0.59) using the conventional method (both *p* < 0.001).

### 3.5. Influence of Filter Pre-Processing

The median AUC values for the five algorithms were not significantly improved by filter pre-processing in either the bi- or single-parameter analyses. No significant improvement with filter pre-processing was observed for any of the three best-performing ML algorithms (i.e., GB, DNN, and RF).

For the conventional method, filter pre-processing significantly improved the AUC values only in the bi-parameter analysis.

## 4. Discussion

The diagnosis of benign or malignant head and neck tumors through pre-treatment imaging is important for subsequent treatment decisions [3,4]; in this context, the clinical utility of ADC maps and DKI [3,4,5,6,19,20,21,22,23,24,25,26,27,28,29] has been reported. This study is the first to report on the use of SDI for the differential diagnosis of benign and malignant head and neck tumors via ML bi-parameter analysis. SDI allows both ADC maps and DKI to be simultaneously acquired in a shorter time than conventional imaging in daily clinical practice; furthermore, the bi-parameter analysis of ADC maps and DKI using ML enables better differential diagnosis of benign and malignant head and neck tumors than conventional methods.

The clinical utility of DWI in the differential diagnosis of benign and malignant head and neck tumors has been reported [3,4,5,6,19,20,22,23,24,25,26,27,28,29]. While there have been few reports on DKI [3,4,19,20,21], the AUC for the differential diagnosis of benign and malignant tumors has been reported in the range of 0.73 [19] to 0.94 [4], reflecting good to very excellent diagnostic performance. On the other hand, there have been many reports on ADC maps [3,4,5,6,19,20,22,23,24,25,26,27,28,29], with AUC ranging from 0.60 [27] to 0.96 [28]; as such, their differential diagnostic performance is satisfactory to very excellent. In the present evaluation of bi-parameter analysis using ML, the AUC was 0.81, and its differential diagnostic ability was excellent, yielding similar results to those reported in the literature.

The most important feature of SDI is that in diagnosis using DWI, both DKI and ADC maps can be obtained in a short time, using general-purpose software, from only ADC maps taken in routine practice. In previous reports, DKI was performed separately from ADC map imaging, taking 4 [3] to 7 [20] minutes, using dedicated software for analysis. The unique feature of the present study is that AUC values almost equivalent to those reported previously, yielding excellent discriminability of benign and malignant tumors, were obtained in a short time and in a simple manner using only SDI.

There have been very few reports on the use of ML multi-parameter analysis for differential diagnosis of the (benign or malignant) status of head and neck tumors. To the best of our knowledge, there are no other reports comparing DKI and multiple ML algorithms for the differential diagnosis of head and neck benign and malignant tumors. Kazerooni et al. [30] have reported that the benign and malignant status of parotid gland tumors can be highly effectively diagnosed through multi-parameter analysis with an SVM using ADC maps and T2-weighted images. Outside of head and neck tumors, there have been several reports demonstrating the improvement in AUC values when combining ML and multi-parameter analysis [31,32,33]. However, none of these reports have provided clear data on the extent to which ML and multi-parameter analysis each improves AUC values. To the best of our knowledge, there is no report that has directly compared the degree of improvement of AUC values when using ML with respect to the conventional method via ROC analysis, considering diagnosis of the (benign or malignant) status of head and neck tumors.

Regarding the usefulness of ML, the degree of improvement of AUC values when using MK values alone was about 12%, increasing from 0.59 (conventional method) to 0.66 (GB). When both ADC and MK values were used together, the highest AUC was 0.81 (GB), comprising a 14% improvement from the conventional method (0.71). In predicting the recurrence of urethral stricture after primary urethroplasty [34], the AUC value of 0.65 for the conventional method was reported to increase by 26% when using ML (to an AUC value of 0.82).

While the median AUC values of the five algorithms considered in this study were all superior to those obtained in the bi-parameter analysis without ML, the algorithms with particularly significantly higher AUC values were GB, DNN, and RF. GB is an algorithm that combines several weak predictive models, such as DTs, to create a strong predictive model, which has been reported to have an excellent AUC (of about 0.85) when diagnosing between benign and malignant lung nodules [35]. RF is an algorithm that combines multiple DTs to reduce overfitting to the training data, which obtained a very excellent AUC (of around 0.99) in diagnosing between liver tumors and cirrhosis [36]. A DNN consists of interconnected neurons organized in a layered fashion, where the output of a given layer is used as the input for the next layer, and has been reported to have an excellent AUC value (of about 0.83) for diagnosing the (benign or malignant) status of sacral tumors [37].

Regarding the usefulness of the multi-parameter analysis presented here, the AUC values obtained with the GB ranged from 0.74 (ADC value alone) and 0.66 (MK value alone) to 0.81 (both together), representing an improvement of 9 and 23%, respectively, when compared to the ADC and MK values alone. In the diagnosis of sleep apnea (SA) [38], Baty et al. have reported a multi-parameter analysis using SVM, yielding AUC values ranging from 0.92 (electrocardiogram (ECG) signal) to 0.98 (ECG signal and breathing frequency signal combined), with the latter comprising an improvement of about 7% compared to the ECG signal alone.

In the present study, there was no improvement due to filter pre-processing. A previous report [14] showed that the reduction in variance of MK values in SDI after filter pre-processing resulted in improved results. In the present study, as we used a larger number of cases, the decrease in variance was not significant for the filtering parameters used, and this may have resulted in the lack of a significant improvement in AUC values.

In this study, due to the small number of cases, we focused on the classification of malignant and benign cases and performed pixel analysis by integrating all pixels in the tumor area [12,13,14]. This approach was chosen because splitting the data at the patient level could result in large variations in the numerical values, which could significantly affect the results. If the number of cases were larger, a validation method in which data partitioning is performed on a case-by-case basis is expected to increase the generalizability of diagnostic performance to unknown cases, but may not guarantee robustness when analyzed with the number of cases in this study.

This study has several limitations. First, the small number of cases and the limited number of lesion types may have introduced selection bias. To improve reliability, the study should be expanded to include more cases and lesion types. Second, although SDI has the advantage of short-time imaging, it has the problem of producing inhomogeneous MK values due to the small amount of information obtained during the short imaging period. Improvement of this problem has been reported using smoothing filter pre-processing [14]; however, although the reported filter parameters were used in this study, the improvements in MK inhomogeneity and AUC values were not significant. For future improvement, optimization of the smoothing filter parameters in clinical practice and improvement of SDI methods may need to be considered. The pixel analysis performed in this study was used as an analysis method for the small number of cases in this study, and future validation is needed to determine how robust it is for unknown patients. Additional new clinical studies using pixel analysis on a case-by-case basis should be considered in the future after increasing the number of cases. Although this study demonstrates the utility of diagnostic methods that rely on sophisticated software and machine learning, ethical considerations may require further clinical research on the reliability of both methods for routine clinical use at this time.

## 5. Conclusions

This clinical study demonstrated that the combination of simple imaging in routine practice using SDI and bi-parameter analysis using ML is useful in improving the differential diagnosis of the benign or malignant status of head and neck tumors. In addition to the qualitative diagnosis of DKI that has been performed in the past, the addition of quantitative diagnosis using this method is expected to improve the accuracy of future diagnoses.

## Figures and Tables

**Table 1:** Case information.

Type (Sex, Mean Age, Range)	Histological Classification (Differentiation or Type)	Number of Cases	Site *	Number of Pixels **
Malignant (M: 9, F: 8, 69, 37–94)	Squamous cell carcinoma	11	Maxilla (4)	434, 334, 219, 132
	(Well: 4		Tongue (4)	289, 245, 198, 21
	Moderately: 2		Mandible (2)	310, 63
	Poorly: 3 Unknown: 2)		Oral floor (1)	302
	Adenoid cyst carcinoma	2	Maxilla	412, 59
	Lymphoma (EBV-positive DLBCL: 1, CD5-positive DLBCL: 1)	2	Maxilla	398, 154
	Osteosarcoma	1	Mandible	117
	Acinic cell carcinoma	1	Maxilla	223
Benign (M: 7, F: 8, 47, 14–80)	Ameloblastoma (Conventional: 3 Unknown: 5)	8	Mandible	1889, 666, 626, 455, 289, 117, 98, 77
	Pleomorphic adenoma	6	Maxilla (4)	419, 162, 105, 37
			Submandibular gland (1)	170
			Upper lip (1)	95
	Dentinogenic ghost cell tumor	1	Maxilla	431

M, males; F, females; EBV, Epstein–Barr virus; DLBCL, diffuse large B-cell lymphoma. * The number in parentheses in Site means the number of cases in each Site. ** The number of pixels in the ROI set for the tumor area of each case.

**Table 2:** Median ADC and MK values without and with filter pre-processing.

	ADC		MK	
	Malignant Median (Q1, Q3)	Benign Median (Q1, Q3)	Malignant Median (Q1, Q3)	Benign Median (Q1, Q3)
Without filter	0.001193 (0.000964, 0.001604)	0.001631 (0.001361, 0.002070)	0.93 (0, 1.43)	0.63 (0.11, 1.04)
With filter	0.001196 (0.000982, 0.001590) *	0.001623 (0.001384, 0.002044) *	0.87 (0.06, 1.36) *	0.65 (0.20, 1.04)

ADC, apparent diffusion coefficient; MK, mean kurtosis; Q1, first quartile; Q3, third quartile. * *p* < 0.05 vs. the variance without filter pre-processing.

**Table 3:** Comparison of the AUC value in the differentiation of benign and malignant tumors.

Method	Algorithm	Without Filter			With Filter		
		ADC&MK	ADC	MK	ADC&MK	ADC	MK
Machine learning	Gradient boosting	0.81 **	0.74	0.66 **	0.80 **	0.75	0.67 **
	Deep neural network	0.80 **	0.73 ^§^	0.66 **	0.79 ** ^§§^	0.74	0.66 **
	Random forest	0.80 **	0.73 ^§^	0.65 ** ^§§^	0.79 ** ^§^	0.74	0.66 **
	Support vector machine	0.79 ** ^§^	0.74	0.65 ** ^§^	0.78 ** ^§§^	0.74	0.65 ** ^§§^
	Decision tree	0.78 ** ^§§§^	0.73	0.66 **	0.77 * ^§§§^	0.74 ^§§^	0.66 **
	Median	0.80 **^1^ **^2 ☨☨1 ☨☨2^	0.73 **^2 ☨☨2 ☨☨3^	0.66 ^☨☨1 ☨☨3^	0.79 ^☨☨1 ☨☨2^	0.74 ^☨☨2^	0.66
Conventional method		0.71 ***^2 ☨1 ☨☨☨2 ☨☨3^	0.72 ***^2 ☨☨☨2 ☨3^	0.59 ^☨☨☨1 ☨2 ☨☨☨3^	0.74 ^☨☨☨2^	0.73 ^☨☨☨2^	0.57

ADC&MK, bi-parameter analysis using ADC and MK; ADC, apparent diffusion coefficient; MK, mean kurtosis; F−, without filter pre-processing; F+, with filter pre-processing. * *p* < 0.05, ** *p* < 0.001 vs. conventional method. ^§^ *p* < 0.05, ^§§^ *p* < 0.01, ^§§§^ *p* < 0.001 vs. gradient boosting. **^1^ *p* < 0.01 vs. F−ADC. **^2^ *p* < 0.01, ***^2^ *p* < 0.001 vs. F−MK. ^☨1^ *p* < 0.05, ^☨☨1^ *p* < 0.01, ^☨☨☨1^ *p* < 0.001 vs. F+ADC. ^☨2^ *p* < 0.05, ^☨☨2^ *p* < 0.01, ^☨☨☨2^ *p* < 0.001 vs. F+MK. ^☨3^ *p* < 0.05, ^☨☨3^ *p* < 0.01, ^☨☨☨3^ *p* < 0.001 vs. F+ADC&MK. Red numbers indicate the AUC values of ML algorithms that showed significantly higher AUC values than the conventional method and the other ML algorithms. Blue numbers indicate the median AUC values among the five ML algorithms for each condition.

**Table 4:** Comparison of AUC values between bi- and single-parameter analyses.

Algorithm	Explanatory Variable	F−ADC&MK	F−ADC	F−MK	F+ADC&MK	F+ADC	F+MK
Gradient boosting	F−ADC&MK	N/A					
	F−ADC	<0.001	N/A				
	F−MK	<0.001	<0.001	N/A			
	F+ADC&MK	NS	<0.001	<0.001	N/A		
	F+ADC	<0.001	NS	<0.001	<0.001	N/A	
	F+MK	<0.001	<0.001	NS	<0.001	<0.001	N/A
Deep neural network	F−ADC&MK	N/A					
	F−ADC	<0.001	N/A				
	F−MK	<0.001	<0.001	N/A			
	F+ADC&MK	NS	<0.001	<0.001	N/A		
	F+ADC	<0.001	NS	<0.001	<0.001	N/A	
	F+MK	<0.001	<0.001	NS	<0.001	<0.001	N/A
Random forest	F−ADC&MK	N/A					
	F−ADC	<0.001	N/A				
	F−MK	<0.001	<0.001	N/A			
	F+ADC&MK	NS	<0.001	<0.001	N/A		
	F+ADC	<0.001	NS	<0.001	<0.001	N/A	
	F+MK	<0.001	<0.001	NS	<0.001	<0.001	N/A

F−, without filter pre-processing; F+, with filter pre-processing; ADC&MK, bi-parameter analysis using ADC and MK; ADC, apparent diffusion coefficient; MK, mean kurtosis; NS, no significant difference; N/A, not applicable. Numbers indicate *p*-values for pairwise permutation tests. Red, blue, and gray colors indicate *p* < 0.001, NS, and N/A, respectively.

## Data Availability

The original contributions presented in this study are included in the article/Supplementary Materials. Further inquiries can be directed to the corresponding author.

## Supplementary Materials

The following supporting information can be downloaded at: https://www.mdpi.com/article/10.3390/diagnostics15060790/s1, Table S1: AIdata_with_filter pre-processing, Table S2: AIdata_without_filter pre-processing, Code S1: GB code with filter pre-processing, Code S2: GB code without filter pre-processing, Hyperparameter S1: GB best hyperparameter with the final validation results with filter pre-processing, Hyperparameter S2: GB best hyperparameter with the final validation results without filter pre-processing.
