# Supplementary material for: Improving Diagnostic Performance for Head and Neck Tumors with Simple Diffusion Kurtosis Imaging and Machine Learning Bi-Parameter Analysis
Source: Diagnostics (Basel). 2025 Mar 20;15(6):790. doi: 10.3390/diagnostics15060790 (PMC11941253; doi:10.3390/diagnostics15060790)
Supplement: Supplementary file 1 [file diagnostics-15-00790-s001.zip › diagnostics-3483560-supplementary/Code S1.pdf]

```
[1]#important libraries
import pandas as pd
import numpy as np

# for splitting and cross validation
from sklearn.model_selection import train_test_split

#algorithms
from sklearn.ensemble import GradientBoostingClassifier

#evaluating the results
from sklearn import metrics
import matplotlib.pyplot as plt
from sklearn.metrics import classification_report
from sklearn.metrics import confusion_matrix
from sklearn.metrics import roc_curve, auc, roc_auc_score

[2]pip install imbalanced-learn

[3]# Oversample and plot imbalanced dataset with SMOTE
from collections import Counter
from imblearn.over_sampling import SMOTE
from matplotlib import pyplot
from numpy import where

[4]# for splitting and cross validation
from sklearn.model_selection import train_test_split
from sklearn.model_selection import RepeatedStratifiedKFold
from sklearn.model_selection import GridSearchCV
from sklearn.model_selection import cross_val_score

[5]import pandas as pd
#Importing Dataset
df = pd.read_excel("C:/Users/hitoy/Desktop/Python/Table S1.xlsx")
print (df)
```

```
#Preparing Data For Training
x = df.drop(columns=["TN"])
x

y = df["TN"]
y

[6]# split data into train and test sets
x_train, x_test, y_train, y_test = train_test_split(x, y, test_size=0.2, random_state=0, stratify=y)

[7]print (y_test)

[8]#Feature Scaling
from sklearn.preprocessing import StandardScaler
sc = StandardScaler()
x_train = sc.fit_transform(x_train)
x_test = sc.transform(x_test)

[9]from imblearn.pipeline import Pipeline, make_pipeline

[10]from sklearn.preprocessing import LabelEncoder
lbl_encoder = LabelEncoder()
y_test= lbl_encoder.fit_transform(y_test)

[11]# summarize class distribution
counter = Counter(y_train)
print(counter)

[12]for k,v in counter.items():
        per = v / len(y) * 100
        print('Class=%d, n=%d (%.3f%%)' % (k, v, per))
# plot the distribution
plt.title('Classes distribution');
pyplot.bar(counter.keys(), counter.values())
pyplot.show()
```

```
[13]#GradientBoosting
from sklearn.ensemble import GradientBoostingClassifier
from imblearn.pipeline import make_pipeline
from imblearn.over_sampling import SMOTE
from sklearn.model_selection import GridSearchCV, RepeatedStratifiedKFold
from sklearn.preprocessing import StandardScaler
from sklearn.metrics import accuracy_score, recall_score, precision_score, f1_score, fbeta_score, confusion_matrix, classification_report, roc_curve, roc_auc_score, cohen_kappa_score, matthews_corrcoef
import matplotlib.pyplot as plt

# Data set partitioning
x_train, x_test, y_train, y_test = train_test_split(x, y, test_size=0.2, random_state=42)

# Pipeline Setup
pipeline = make_pipeline(
    SMOTE(sampling_strategy='auto', random_state=0),
    StandardScaler(),
    GradientBoostingClassifier(random_state=0)
)

# Grid settings for hyperparameters
param_grid = {
    'gradientboostingclassifier__n_estimators': [50, 100, 200],
    'gradientboostingclassifier__learning_rate': [0.01, 0.1, 0.5],
    'gradientboostingclassifier__max_depth': [3, 4, 5],
    'gradientboostingclassifier__subsample': [0.6, 0.7, 0.8, 0.9, 1.0],
}

# Hyperparameter optimization with RepeatedStratifiedKFold
cv = RepeatedStratifiedKFold(n_splits=5, n_repeats=5, random_state=0)
grid_search = GridSearchCV(pipeline, param_grid, cv=cv, scoring='roc_auc', n_jobs=-1)
grid_search.fit(x_train, y_train)

# Display of optimal parameters
```

```
print('Best parameters found: ', grid_search.best_params_)
print('Best cross-validation AUC score: ', grid_search.best_score_)

# Prediction with the best model
best_model = grid_search.best_estimator_
y_pred = best_model.predict(x_test)
y_proba = best_model.predict_proba(x_test)[:, 1]

# Calculation of various evaluation indices
accuracy = accuracy_score(y_test, y_pred)
precision = precision_score(y_test, y_pred)
recall = recall_score(y_test, y_pred)
f1 = f1_score(y_test, y_pred)
conf_matrix = confusion_matrix(y_test, y_pred)
specificity = conf_matrix[0, 0] / (conf_matrix[0, 0] + conf_matrix[0, 1])
kappa = cohen_kappa_score(y_test, y_pred)
mcc = matthews_corrcoef(y_test, y_pred)

print('* Accuracy Score :', accuracy)
print('* Precision Score :', precision)
print('* Recall Score :', recall)
print('* F1 Score :', f1)
print("Specificity:", specificity)
print("Cohen's Kappa:", kappa)
print("Matthews Correlation Coefficient:", mcc)

# Calculation of ROC-AUC
fpr, tpr, thresholds = roc_curve(y_test, y_proba)
plt.plot(fpr, tpr, marker='o')
plt.xlabel('FPR: False positive rate')
plt.ylabel('TPR: True positive rate')
plt.grid()
ROC_AUC = roc_auc_score(y_test, y_proba)
print('ROC_AUC score:', ROC_AUC)

import pandas as pd
```

```
# Store test data features, prediction results, and actual target values in DataFrame
final_test_data = pd.DataFrame(x_test, columns=["MK", "ADC"])    # Rename columns MK
and ADC
final_test_data["Predicted_Target"] = y_pred
final_test_data["Predicted_Probability"] = y_proba
final_test_data["Target"] = y_test

# Output DataFrame as CSV file
final_test_data.to_csv("final_test_results07y.csv", index=False)

# Show link for download
print("Final test results have been saved to 'final_test_results07y.csv'.")

from IPython.display import FileLink

FileLink("final_test_results07y.csv")
```
