# Supplementary material for: Improving Diagnostic Performance for Head and Neck Tumors with Simple Diffusion Kurtosis Imaging and Machine Learning Bi-Parameter Analysis
Source: Diagnostics (Basel). 2025 Mar 20;15(6):790. doi: 10.3390/diagnostics15060790 (PMC11941253; doi:10.3390/diagnostics15060790)
Supplement: Supplementary file 1 [file diagnostics-15-00790-s001.zip › diagnostics-3483560-supplementary/Hyperparameter S2.pdf]

Best parameters
gradientboostingclassifier__learning_rate: 0.01
gradientboostingclassifier__max_depth: 5
gradientboostingclassifier__n_estimators: 200
gradientboostingclassifier__subsample: 0.6

The final validation of the predictions against the test data
ROC_AUC score: 0.81
Accuracy Score : 0.74
Precision Score : 0.69
Recall Score : 0.67
F1 Score : 0.68
Specificity: 0.79
Cohen's Kappa: 0.46
Matthews Correlation Coefficient: 0.46
